# Deterministic Sensing Matrices in Compressive Sensing: A Survey

**DOI:** 10.1155/2013/192795

**Published:** 2013-11-05

**Authors:** Thu L. N. Nguyen, Yoan Shin

**Affiliations:** School of Electronic Engineering, Soongsil University, Seoul 156-743, Republic of Korea

## Abstract

Compressive sensing is a sampling method which provides a new approach to efficient signal compression and recovery by exploiting the fact that a sparse signal can be suitably reconstructed from very few measurements. One of the most concerns in compressive sensing is the construction of the sensing matrices. While random sensing matrices have been widely studied, only a few deterministic sensing matrices have been considered. These matrices are highly desirable on structure which allows fast implementation with reduced storage requirements. In this paper, a survey of deterministic sensing matrices for compressive sensing is presented. We introduce a basic problem in compressive sensing and some disadvantage of the random sensing matrices. Some recent results on construction of the deterministic sensing matrices are discussed.

## 1. Introduction

 Consider a scenario that **x** ∈ ℝ^*n*^ is a vector we wish to recover. Let **y** ∈ ℝ^*m*^  (*m* ≪ *n*) be a linear measurements of the vector **x**, which is given by

(1) $$\begin{array}{c} y=Ax, \end{array}$$

where **A** is the measurement matrix or sensing matrix. Because this system is underdetermined, the recovery problem of the vector **x** from the measurement vector **y** is an ill-posed problem. However, two papers by Donoho [1] and Candès et al. [2] gave us a breakthrough by exploiting sparsity in recovery problems. The authors show that a sparse signal can be reconstructed from very few measurements by solving via *l*_0_-minimization

(P_0_) $$\begin{array}{c} \underset{z\in {\mathbb{R}}^{n}}{\min}{||z||}_{0}\;\text{subject}\;\;\text{to}\;\;Az=y, \end{array}$$

*l*_1_-minimization

(P_1_) $$\begin{array}{c} \underset{z\in {\mathbb{R}}^{n}}{\min}{||z||}_{1}\;\text{subject}\;\;\text{to}\;\;Az=y, \end{array}$$

or adopting a strategy between ((P_0_)) and ((P_1_))

(P_q_) $$\begin{array}{c} \underset{z\in {\mathbb{R}}^{n}}{\min}{||z||}_{q}\;\text{subject}\;\;\text{to}\;\;Az=y. \end{array}$$

The sufficient conditions for having the solution of ((P_0_)) to coincide with that of ((P_1_)) are dependent on either mutual coherence or Restricted Isometry Property (RIP). These conditions are closely related to each other and play an important role in the construction of sensing matrices. Consider **A** = [**a**_1_ ⋯ **a**_*n*_] is an *m* × *n* sensing matrix we investigate. Then its mutual coherence is defined as

(2) $$\begin{array}{c} \mu \left(A\right)=\max\frac{\left|\langle a_{i},a_{j}\rangle \right|}{{||a_{i}||}_{2}{||a_{j}||}_{2}},\;i,j=1,\ldots ,n. \end{array}$$

**Lemma 1 (see [3])**

For an *m* × *n* sensing matrix **A**, the Welch bound is given by

(3) $$\begin{array}{c} \sqrt{\frac{n-m}{m\left(n-1\right)}}\leq \mu \left(A\right)\leq 1. \end{array}$$

The existence and uniqueness of the solution can be guaranteed as soon as the measurement matrix **A** satisfies the RIP of order *k*; that is,

(4) $$\begin{array}{c} \left(1-\delta_{k}\right){||z||}_{2}^{2}\leq {||Az||}_{2}^{2}\leq \left(1+\delta_{k}\right){||z||}_{2}^{2},\;\text{where}\;\;{||z||}_{0}\leq k. \end{array}$$

The smallest value of *δ*_*k*_ is called the Restricted Isometry Constant (RIC). A strict condition *δ*_2*s*_ < 1 also guarantees exact solution via *l*_0_-minimization. However, the problem ((P_0_)) remains NP-hard; that is, it cannot be solved in practice. For 0 < *q* ≤ 1, there is no numerical scheme to compute solutions with minimal *l*_*q*_-norm as well. Furthermore, the problem ((P_1_)) is a convex optimization problem, and in fact, it can be formulated as a linear optimization problem. Then solving via *l*_1_-minimization is efficient with high probability. Hence, most researchers are interested in the recovery via *l*_1_-minimization.

There are two common ways to solve these problems. First, we can exactly recover **x** via *l*_1_-minimization by solving the problem ((P_1_)) or ((P_1,*ϵ*_)) which is given as

(P_1,ϵ_) $$\begin{array}{c} \underset{z\in {\mathbb{R}}^{n}}{\min}{||z||}_{1}\;\text{subject}\;\;\text{to}\;\;{||Az-y||}_{2}\leq \epsilon . \end{array}$$

The second method is using greedy algorithms for *l*_0_-minimization, such as Matching Pursuit (MP), Orthogonal Matching Pursuit (OMP), or their modifications [4–8].

However, in order to ensure unique and stable reconstruction, the sensing matrix **A** must satisfy some criteria. One of the well-known criteria is 2*k*-RIP. More attention has been paid to random sensing matrices generated by identical and independent distributions (i.i.d.) such as Gaussian, Bernoulli, and random Fourier ensembles, to name a few. Their applications have been shown in medical images processing [9], geophysical data analysis [10], communications [11, 12], and other various signal processing problems. Even though random sensing matrices ensure high probability in reconstruction, they also have many drawbacks such as excessive complexity in reconstruction, significant space requirement for storage, and no efficient algorithm to verify whether a sensing matrix satisfies RIP property with small RIC value. Hence, exploiting specific structures of deterministic sensing matrices is required to solve these problems of the random sensing matrices.

Recently, several deterministic sensing matrices have been proposed. We can classify them into two categories. First are those matrices which are based on coherence [13–15]. Second are those matrices which are based on RIP or some weaker RIPs [16–20]. In this paper, we introduce some highlighted results such as deterministic construction of sensing matrices via algebraic curves over finite fields in term of coherence and chirp sensing matrices, second-order Reed-Muller codes, binary Bose-Chaudhuri-Hocquenghem (BCH) codes, and the sensing matrices with statistical RIP in terms of the RIP.

The rest of this paper is organized as follows.  Section 2 introduces some random sensing matrices and their practical disadvantages. In Section 3, we present some highlighted results in terms of deterministic constructions.  Section 4 concludes this paper.

## 2. Random Sensing Matrices and Their Drawbacks

 Recall that **x** ∈ ℝ^*n*^ is the vector we want to recover. Because the number of measurements is much smaller than its dimension (*m* ≪ *n*), we cannot find a linear identity reconstruction map; that is, unique solution does not exist for all **x** in ℝ^*n*^. However, if we assume that the signal **x** belongs to a certain subset Σ_*k*_ ⊂ ℝ^*n*^ which is the set of all *k*-sparse vectors as

(5) $$\begin{array}{c} \Sigma_{k}\left(x\right)=\left\{x\in {\mathbb{R}}^{n}:x_{i}=0,i\notin T\right\} \end{array}$$

for each index set *T*⊆{1,…, *n*}, the *k*-best approximation is given by

(6) $$\begin{array}{c} \sigma_{k}{\left(x\right)}_{q}=\inf\left\{{||x-z||}_{q},{||z||}_{0}\leq k\right\}, \end{array}$$

where ||·||_*q*_ can be any norm in **R** ^*n*^. As we noted above, the use of randomly generated sensing matrices has become powerful in compressive sensing. For an upper bound

(7) $$\begin{array}{c} k\leq \frac{cm}{\log\left(n/m\right)}, \end{array}$$

where *c* is a positive constant, the i.i.d. Gaussian matrix achieves the *k*-RIP as well, which guarantees to recover sparse signals with high probability [21, 22]. The condition in (7) is also known to hold for the symmetric Bernoulli distribution case and changed to *k* ≤ *cm*/(log⁡⁡*n*)^6^ for the Fourier measurements [23]. For noiseless recovery, it can be stated as follows.

**Theorem 2 (see [24])**

If **z** ∈ ℝ^*n*^ is a *k*-sparse vector and the sensing matrix **A** satisfies

(8) $$\begin{array}{c} \delta_{k}\left(A\right)+\delta_{2k}\left(A\right)+\delta_{3k}\left(A\right)<1, \end{array}$$

then **x** is the unique minimizer to ((P_1_)).

In practice, the original signals may be affected by noise, so the recovered signals are not exact, and rather they are almost sparse instead. Hence, some modified criteria were proposed as follows.

**Theorem 3 (see [25])**

Suppose that **x** ∈ Σ_*k*_ and the noise **e** = **A** **z** − *y* satisfies ||**e**||_2_ ≤ *ϵ*. If the sensing matrix **A** has RIP such that

(9) $$\begin{array}{c} \delta_{3k}\left(A\right)+3\delta_{4k}\left(A\right)<2, \end{array}$$

then x* which is the output of the reconstruction algorithm applied to **x** via ((P_1,*ϵ*_)) will obey

(10) $$\begin{array}{c} {||x^{*}-x||}_{2}\leq c\epsilon , \end{array}$$

where the constant *c* depends on sparsity *k*.

A new result on RIC was proposed by Candès as follows.

**Theorem 4 (see [25])**

Given **x** ∈ Σ_*k*_(**x**) and an upper bound of noise ||**e**||_2_ = ||**A** **z**−**y**||_2_ ≤ *ϵ*, if the sensing matrix **A** has RIP such that

(11) $$\begin{array}{c} \delta_{2k}<\sqrt{2}-1, \end{array}$$

then any solution x* of ((P_1,*ϵ*_)) obeys

(12) $$\begin{array}{c} {||x^{*}-x||}_{2}\leq c_{1}\epsilon +c_{2}\frac{\sigma_{k}{\left(x\right)}_{2}}{\sqrt{k}}, \end{array}$$

where *c*_1_ and *c*_2_ are two positive constants depending on sparsity *k*.

Several inequalities in terms of RIC have been discovered, such as $\delta_{2k}<2/(3+\sqrt{2})$ in [26], $\delta_{2k}<3/(4+\sqrt{6})$ in [27], or $\delta_{2k}<2/(2+\sqrt{5})$ in [28], to name a few. In sum, we can obtain stable and unique solution by using tools from random sensing matrices.

Random matrices are easy to construct and ensure high probability reconstruction. However, they also have many drawbacks. First, storing random matrices requires a lot of storage. Second, there is no efficient algorithm verifying RIP condition. So far, it is not a good approach because of its lack of efficiency. The recovery problems may be difficult when the dimension of the signal becomes large, and we have to construct a measurement matrix that satisfies RIP with a small *δ*_*k*_, such as Theorem 4.

## 3. Deterministic Sensing Matrices

### 3.1. Chirp Sensing Matrices

 A discrete chirp of length *m* has the form

(13) $$\begin{array}{c} A_{r,\omega }=\frac{1}{\sqrt{m}}\exp\left\{\frac{2\pi i}{m}\omega l+\frac{2\pi i}{m}rl^{2}\right\},\;l,\omega ,r\in {\mathbb{Z}}_{m}, \end{array}$$

where *r* is the chirp rate and *ω* is the base frequency. The full chirp sensing matrix **A** of size *m* × *m* ^2^ can be written as

(14) $$\begin{array}{c} A_{\text{chirp}}=\left[\begin{array}{cccc} U_{r_{1}} & U_{r_{2}} & \cdots & U_{r_{k}} \end{array}\right]. \end{array}$$

Each matrix **U**_*r*_*t*__  (*t* = 1,…, *ω*) is an *m* × *m* matrix with columns given by the chirp signals having a fixed chirp rate *r*_*t*_ with base frequency *ω* varying from 0 to *m* − 1. For instance, given *k* = 2 and *r*, *m*, *l* ∈ {0,1}, we obtain

(15) $$\begin{array}{c} U_{r_{1}}=\left[\begin{array}{cc} 1 & 1\\ 1 & e^{i\pi } \end{array}\right],\;\;U_{r_{2}}=\left[\begin{array}{cc} 1 & 1\\ e^{i\pi } & e^{i\pi +i2\pi } \end{array}\right]. \end{array}$$

Hence, we get the 2 × 4 deterministic sensing matrix **A** as

(16) $$\begin{array}{c} A_{\text{chirp}}=\left[\begin{array}{cc} U_{r_{1}} & \;U_{r_{2}} \end{array}\right]=\left[\begin{array}{cccc} 1 & 1 & 1 & 1\\ 1 & e^{i\pi } & e^{i\pi } & e^{i\pi +i2\pi } \end{array}\right]. \end{array}$$

Note that when *r* = *r*_1_ = 0, the matrix **U**_*r*_1__ corresponding to chirp rate *r*_1_ becomes the Discrete Fourier Transform (DFT) matrix.

Most of the sensing chirp matrices admit a fast reconstruction algorithm which reduces the complexity to *O*(*km*log⁡⁡*m*).

### 3.2. Second-Order Reed-Muller Sensing Matrices

 The second-order Reed-Muller code is given as follows:

(17) $$\begin{array}{c} \phi_{P,b}\left(a\right)=\frac{{\left(-1\right)}^{w(b)}}{\sqrt{2^{p}}}i^{{\left(2b+Pa\right)}^{T}a}, \end{array}$$

where **P** is a *p* × *p* binary symmetric matrix, **b** is a *p* × 1 binary vector in *ℤ*_*p*_^2^, and *w*(**b**) is the weight of **b**, that is, number of bit-1 entries. In practice, the matrices **P** are set as all-zero matrices or the matrices with zero diagonals. Thus, there are only 2^*p*(*p*−1)/2^ matrices **P** satisfying this condition, which are {**P**_1_,…, **P**_2^*p*(*p*−1)/2^_}, and the functions {*ϕ*_**P**,**b**_(**a**)} are real valued. The set

(18) $$\begin{array}{c} {ℱ}_{P}=\left\{\phi_{P,b}\;|\;b\in {\mathbb{Z}}_{2}^{p}\right\} \end{array}$$

forms a basis of *ℤ*_2_^*p*^. The inner product on *ℱ*_**P**_ is defined as follows. For any two vectors *ϕ*_**P**,**b**_ and *ϕ*_**P**′,**b**′_ in *ℱ*_**P**_,

(19) $$\begin{array}{c} \langle \phi_{P,b},\phi_{P^{\prime },b^{\prime }}\rangle =\{\begin{array}{cc} \frac{1}{\sqrt{2^{q}}} & 2^{q}\;\;\text{times},\\ 0 & 2^{p}-2^{q}\;\;\text{times}, \end{array} \end{array}$$

where *q* = rank⁡(**P** − **P**′). The deterministic sensing matrix has the form

(20) $$\begin{array}{c} A_{\text{RM}}={\left[\begin{array}{cccc} U_{P_{1}} & U_{P_{2}} & \cdots & U_{P_{2^{p(p-1)/2}}} \end{array}\right]}_{2^{p}\times 2^{p(p+1)/2}}, \end{array}$$

where **U**_**P**_**i**__ is the unitary matrix corresponding to *ℱ*_*P*_*i*__  (*i* = 1,…, 2^*p*(*p*−1)/2^). Note that if we set *m* = 2^*p*^ and *n* = 2^*p*(*p*+1)/2^, we get an *m* × *n* sensing matrix **A**_RM_. For instance, let *p* = 2; then

(21) $$\begin{array}{c} {\mathbb{Z}}_{2}^{2}=\left\{\left[\begin{array}{c} 0\\ 0 \end{array}\right],\left[\begin{array}{c} 0\\ 1 \end{array}\right],\left[\begin{array}{c} 1\\ 0 \end{array}\right],\left[\begin{array}{c} 1\\ 1 \end{array}\right]\right\}. \end{array}$$

There are only 2^2(2−1)/2^ = 2 binary symmetric matrices **P** of size 2 × 2 satisfying the condition. These are

(22) $$\begin{array}{c} P_{1}=\left[\begin{array}{cc} 0 & 0\\ 0 & 0 \end{array}\right],\;\;P_{2}=\left[\begin{array}{cc} 0 & 1\\ 1 & 0 \end{array}\right]. \end{array}$$

Hence, we get the deterministic sensing matrix **A** as

(23) ARM=[UP1UP2]=[1−1−111−1−1111−1−111−1−11−11−11−11−11111−1−1−1−1]22×23.

Reconstruction algorithms using the second-order Reed-Muller sensing matrices can outperform the standard compressive sensing using random matrices via *l*_1_-minimization, especially when the original signal is not sparse and the noise is present. Moreover, the nesting of the Delsarter-Goethals sets of the Reed-Muller codes is still feasible if the dimension of the original signal is large [17, 29]. 

### 3.3. Binary BCH Matrices

 Denote *n* as a divisor of 2^*p*^ − 1 for some integer *p* ≥ 3, and *γ* ∈ GF(2^*p*^) as a primitive *n*th root of unity and assume that *p* is the smallest integer for which *n* divides 2^*p*^ − 1. If we set *α* = *γ* ^(2^*p*^−1)/*n*^, then *α* has order *n*. Define a code *𝒞* over GF(2^*p*^) by

(24) $$\begin{array}{c} H={\left[\begin{array}{ccccc} 1 & \alpha & \alpha^{2} & \cdots & \alpha^{\left(n-1\right)}\\ 1 & \alpha^{3} & \alpha^{6} & \cdots & \alpha^{3(n-1)}\\ \vdots & \vdots & \vdots & \vdots & \vdots \\ 1 & \alpha^{2i-1} & \alpha^{2(2i-1)} & \cdots & \alpha^{\left(n-1)(2i-1\right)}\\ \vdots & \vdots & \vdots & \vdots & \vdots \\ 1 & \alpha^{2l-1} & \alpha^{2(2l-1)} & \cdots & \alpha^{\left(n-1)(2l-1\right)} \end{array}\right]}_{l\times n}. \end{array}$$

The BCH code *𝒞* is defined by

(25) $$\begin{array}{c} C=\left\{c\in {𝔽}_{2^{n}}\;|\;H\cdot c^{†}=0\right\}. \end{array}$$

In other words, if we denote *𝒩* as the null-space of the above matrix of GF(2^*p*^), then the BCH code *𝒞* = *𝒩*∩*𝔽*_2^*n*^_.

An example of binary matrices formed by BCH code is given as follows. Let *n* = 15, *p* = 4, and *t* = 1, and let *γ* be a primitive element of GF(2^4^) = GF(16) satisfying *γ* ^4^ + *γ* + 1 = 0. Then *α* = *γ* ^(2^*p*^−1)/*n*^ = *γ* ^(2^4^−1)/15^ = *γ*. The BCH code is the set of 15 tuples that lie in the null space of the matrix

(26) $$\begin{array}{c} H=\left[\begin{array}{ccccc} 1 & \alpha & \alpha^{2} & \cdots & \alpha^{14} \end{array}\right]. \end{array}$$

Since

(27) g(x)=(x4+x+1)(x4+x3+x2+x+1)=x8+x7+x6+x4+1

satisfies *g*(*α* ^3^) = 0, we have [1 0 0 0 1 0 1 1 1 0 0 0 0 0 0]^*T*^ as a codeword in the BCH code. The binary matrix is obtained as follows:

(28) Abin=[0 0 0 1 0 0 1 1 0 1 0 1 1 1 10 0 1 0 0 1 1 0 1 0 1 1 1 1 00 1 0 0 1 1 0 1 0 1 1 1 1 0 01 0 0 0 1 0 0 1 1 0 1 0 1 1 1].

Since BCH code is cyclic, we can describe it in terms of a generator polynomial which is the smallest degree polynomial having zeros *α*, *α* ^3^,…, *α* ^2*i*−1^,…, *α* ^2*l*−1^. The advantages of these matrices are deterministic construction, simplicity in sampling process, and reduced computational complexity compared with the DeVoice's binary sensing matrices. However, the generated matrices formed by BCH codes do not acchieve the RIP constraint yet.

### 3.4. Sensing Matrices with Statistical Restricted Isometry Property

 In [18], the authors proposed some weaker Statistical Restricted Isometry Properties (StRIPs) defined as follows.

**Definition 5 (StRIP)**

An *m* × *n* matrix *A* is called StRIP of order *k* with constant *δ* and probability 1 − *ϵ* if

(29) $$\begin{array}{c} \text{Pr}\left({||Ax||}^{2}-{||x||}^{2}\right)\geq 1-\epsilon \end{array}$$

with respect to a uniform distribution of vector **x** among all *k*-sparse vectors in **R** ^*n*^ of the same norm.

**Definition 6 (UStRIP)**

An *m* × *n* matrix *A* is called UStRIP of order *k* with constant *δ* and probability 1 − *ϵ* if it satisfies StRIP and

(30) $$\begin{array}{c} \left\{z\in {\mathbb{R}}^{n}:Az=Ax\right\}=\left\{x\right\} \end{array}$$

with probability exceeding 1 − *ϵ* with respect to a uniform distribution of vector **x** among all *k*-sparse vectors in **R** ^*n*^ of the same norm.

These constructions allow recovery methods for which expected performance is sublinear in *n* and quadratic in *m*, compared to the superlinear in *n* of the BP or the MP algorithms. The criteria are simple; however, when satisfied by a deterministic sensing matrix, they guarantee successful recovery in an exponentially small fraction of *k*-sparse signals. The authors also showed that such sensing matrices satisfying these aforementioned properties could be constructed by chirps, second-order Reed-Muller codes, and BCH codes [16–18].

### 3.5. Deterministic Construction of Sensing Matrices via Algebraic Curves over Finite Fields

 In [14], DeVore used polynomials over finite field *𝔽*_*p*_ to construct binary sensing matrices of size *p* ^2^ × *p* ^*k*+1^, where *p* is a primer number. Let {*a*_0_, *a*_1_,…, *a*_*r*_} be a subset of *𝔽*_*q*_, and let *P*_*r*_ be the set of all polynomials of degree no more than *r* on *𝔽*_*p*_ as

(31) $$\begin{array}{c} P_{r}=\left\{f\in {𝔽}_{p}\left[x\right]:\deg f\leq k\right\}. \end{array}$$

There are *n* = *p* ^*r*+1^ such polynomials. Any polynomial *P* ∈ *P*_*r*_ can be described as a mapping

(32) $$\begin{array}{c} P:{𝔽}_{p}\to F_{p},\\ \left(x^{0},x^{1},\ldots ,x^{r}\right)\mapsto P\left(x\right)=a_{0}x^{0}+a_{1}x^{1}+\cdots +a_{r}kx^{r}. \end{array}$$

The set of (**x**, *P*(**x**)) is a subset of *𝔽*_*p*_ × *𝔽*_*p*_. We order the element of *𝔽*_*p*_ × *𝔽*_*p*_ as (0,0), (0,1),…, (*p* − 1, *p* − 1). For given *𝔽*_*p*_, the set *𝔽*_*p*_ × *𝔽*_*p*_ has *m* = *p* ^2^ of order pairs. For each *P* ∈ *P*_*r*_, we denote **v**_*P*_ as the vector indexed on *𝔽*_*p*_ × *𝔽*_*p*_ which is defined by

(33) $$\begin{array}{c} {\left[P_{0,0},\ldots ,P_{0,p-1},P_{1,0},\ldots ,P_{1,p-1},\ldots ,P_{p-1,0},\ldots ,P_{p-1,p-1}\right]}^{T}, \end{array}$$

where

(34) $$\begin{array}{c} P_{i,j}=\{\begin{array}{cc} 1, & \text{if}\;\;P\left(i\right)=j,\\ 0, & \text{otherwise} \end{array} \end{array}$$

for *i*, *j* = 0,1,…, *p* − 1.

**Theorem 7**

Let **A**_0_ be the *m* × *n* matrices with columns **v**_*P*_, *P* ∈ *P*_*r*_ with these columns ordered lexicographically with respect to the coefficients of the polynomial. Then the matrix $A=\left(1/\sqrt{p}\right)A_{0}$ satisfies the RIP of order *k* < *p*/*r* + 1 with RIC value *δ*_*k*_ = (*k* − 1)*r*/*p*.

There are several deterministic constructions of sensing matrices via algebraic curves over finite fields called algebraic geometry codes [30–33]. Goppa's code is one of well-known results which contain many linear codes with many good parameters. Hence, these kinds of sensing matrices are good candidates in reconstruction issues using compressive sensing.

### 3.6. Binary Sensing Matrices Generated by Unbalanced Expander Graphs

 In [20], a large class of deterministic sensing matrices based on unbalanced expander graphs, that is, the combinatorial structures, was proposed. Denoting [*n*] = {1 ⋯ *n*}, these bipartite graphs are formalized through the following definitions.

**Definition 8**

A bipartite graph with *n* left vertices, *m* right-vertices, and left-degree *d* is specified by a function Γ : [*n*]×[*d*]→[*m*], where Γ(*x*, *y*) denotes the *y*th neighbor of *x*. For a set *S* ⊂ [*N*], we write Γ(*S*) to denote its set of neighbors {Γ(*x*, *y*) : *x* ∈ *S*,  *y* ∈ [*D*]}.

**Definition 9**

A bipartite graph Γ : [*n*]×[*d*]→[*m*] is a (*K*, *A*) expander if for every set *S* ⊂ [*n*] of size *k*, we have |Γ(*S*)|≥*A* · *K*.

They constructed a large class of binary and sparse matrices satisfying a different form of the RIP property called RIP-*p* as

(35) (1−δk)||z||pp≤||Az||pp≤(1+δk)||z||pp, where  ||z||0≤k.

If the sensing matrix **A** is an adjacency matrix of high-quality unbalanced expander, then the RIP-*p* holds for 1 ≤ *p* ≤ 11 + *O*(1)/log⁡(*n*).

**Theorem 10 (see [19])**

Consider any *m* × *n* matrix **A**_0_ which is the adjacency matrix of an (*k*, *ϵ*) unbalanced expander *G* = (*A*, *B*, *E*), |*A* | = *n*, |*B* | = *m* with left degree *d*, such that 1/*ϵ* and *d* < *n*. Then the scaled matrix $A=\left(1/\sqrt{d}\right)A_{0}$ satisfies the RIP with RIC value *δ* = *cϵ* for some positive constant *c* > 1.

This approach utilizes sparse matrices interpreted as adjacency matrices of sparsity to recover an approximation to the original signal. The new property RIP-*q* suffices to guarantee exact recovery algorithms.

## 4. Concluding Remarks

 In this paper, various deterministic sensing matrices have been investigated and presented in terms of coherence and RIP. The advantages of these matrices, in addition to their deterministic constructions, are the simplicity in sampling and recovery process as well as small storage requirement. It can be possible to make further improvement in both reconstruction efficiency and accuracy using these deterministic matrices in compressive sensing, particularly when some *a priori* information on location of nonzero components is available. 
